# Wave-shaped microfluidic chip assisted point-of-care testing for accurate and rapid diagnosis of infections

**DOI:** 10.1186/s40779-022-00368-1

**Published:** 2022-02-11

**Authors:** Bin-Feng Yin, Xin-Hua Wan, Ming-Zhu Yang, Chang-Cheng Qian, A. S. M. Muhtasim Fuad Sohan

**Affiliations:** 1 School of Mechanical Engineering, Yangzhou University, Yangzhou, 225127 Jiangsu China; 2 Department of Biochemistry, Institute of Basic Medical Sciences, Chinese Academy of Medical Sciences (CAMS) and Peking Union Medical College, Beijing, 100005 China

**Keywords:** Point-of-care testing (POCT), Infection markers, Wave-shaped microfluidic chip, Chemiluminescence, Multiplex detection

## Abstract

**Background:**

Early diagnosis and classification of infections increase the cure rate while decreasing complications, which is significant for severe infections, especially for war surgery. However, traditional methods rely on laborious operations and bulky devices. On the other hand, point-of-care (POC) methods suffer from limited robustness and accuracy. Therefore, it is of urgent demand to develop POC devices for rapid and accurate diagnosis of infections to fulfill on-site militarized requirements.

**Methods:**

We developed a wave-shaped microfluidic chip (WMC) assisted multiplexed detection platform (WMC-MDP). WMC-MDP reduces detection time and improves repeatability through premixing of the samples and reaction of the reagents. We further combined the detection platform with the streptavidin–biotin (SA-B) amplified system to enhance the sensitivity while using chemiluminescence (CL) intensity as signal readout. We realized simultaneous detection of C-reactive protein (CRP), procalcitonin (PCT), and interleukin-6 (IL-6) on the detection platform and evaluated the sensitivity, linear range, selectivity, and repeatability. Finally, we finished detecting 15 samples from volunteers and compared the results with commercial ELISA kits.

**Results:**

Detection of CRP, PCT, and IL-6 exhibited good linear relationships between CL intensities and concentrations in the range of 1.25–40 μg/ml, 0.4–12.8 ng/ml, and 50–1600 pg/ml, respectively. The limit of detection of CRP, PCT, and IL-6 were 0.54 μg/ml, 0.11 ng/ml, and 16.25 pg/ml, respectively. WMC-MDP is capable of good adequate selectivity and repeatability. The whole detection procedure takes only 22 min that meets the requirements of a POC device. Results of 15 samples from volunteers were consistent with the results detected by commercial ELISA kits.

**Conclusions:**

WMC-MDP allows simultaneous, rapid, and sensitive detection of CRP, PCT, and IL-6 with satisfactory selectivity and repeatability, requiring minimal manipulation. However, WMC-MDP takes advantage of being a microfluidic device showing the coefficients of variation less than 10% enabling WMC-MDP to be a type of point-of-care testing (POCT). Therefore, WMC-MDP provides a promising alternative to POCT of multiple biomarkers. We believe the practical application of WMC-MDP in militarized fields will revolutionize infection diagnosis for soldiers.

**Supplementary Information:**

The online version contains supplementary material available at 10.1186/s40779-022-00368-1.

## Background

Infections can lead slight skin irritation to severe organ failure if left untreated, especially in the fields of trauma and war surgery. Bacterial and viral infections are the main types of infection [1, 2]. Severe infections can result in sepsis. Delaying an hour in sepsis treatment increases the risk of death by 6–10% [3]. Therefore, it is necessary to analyze biomarkers for rapid, accurate diagnosis and classification of infections [4–6]. Multiplex detection of C-reactive protein (CRP) [7], procalcitonin (PCT) [8], and interleukin-6 (IL-6) [9] is a reliable method for accurately diagnosing infections and sepsis [10]. In normal human serum, the content of CRP is generally less than 8 μg/ml. The elevation of CRP has a positive correlation with the severity of infection [11]. High levels of CRP are related to bacterial infections and some viral infections like Hemagglutinin 1 Neuraminidase 1 (H1N1) and Corona Virus Disease 2019 (COVID-19) [12–14]. Coronary heart disease [15, 16], cancer [17, 18], and other diseases of such kind have high concentrations of CRP. For this reason, diagnoses of infections relying on the detection of CRP are not accurate. Normally, level of PCT in blood is under 0.5 ng/ml for healthy adults. PCT is a specific index for detection of bacterial infection [19]. The concentration remains normal when viral infections occur. IL-6 is a highly sensitive and specific biomarker for diagnosis of infections. The level of IL-6 in normal human serum is less than 75 pg/ml [20], which is extremely low. It is hard to detect IL-6 for identifying viral and bacterial infections. In serums of patients with severe infections or sepsis, levels of IL-6 exceed 1000 pg/ml. CRP, PCT, and IL-6 have different abnormal times and characteristics. It may lead to misdiagnosis of infections if only to detect a single biomarker. Multiplexed detections of CRP, PCT, and IL-6 can cover the whole period of infection. It effectively assesses the classification and severity of infections [21]. It is imperative to develop a multiplexed detection technique for infectious biomarkers in the fields of militarized point-of-care testing (POCT) [22–24].

As an emerging platform for medical detection [25–27], microfluidic chips have the potential to develop militarized POCT devices [28, 29]. Mou et al. [30] achieved multiplexed detection of CRP, PCT, and IL-6 by using electrospun microfibers and gold nanoparticles to amplify signals. But the procedures of detection cost more than an hour. Additionally, laborious operations and instability made it difficult to be applied in complex environments like battlefields. Russell et al. [31] detected IL-6 in 2.5 min with electro-chemiluminescence (ECL) immunoassay. However, it can hardly enable classificatory diagnosis because of its limitation of a single biomarker and low sensitivity. Zupančič et al. [32] described a low-cost electrochemical sensor platform for simultaneously detecting multiple sepsis biomarkers in whole blood. They incorporated a nanocomposite coating made of cross-linked bovine serum albumin to prevent biofouling while maintaining electroconductivity. It is a potential platform to integrate detection of the three biomarkers in one sensor system.

Here, we develop a wave-shaped microfluidic chip (WMC) assisted multiplexed detection platform (WMC-MDP) for CRP, PCT, and IL-6 in conjunction with a micromixer, chemiluminescence (CL), and streptavidin–biotin (SA-B) system. WMC-MDP has a sandwich structure. WMC consists of a channel layer above, a base layer below, and a detection layer in the middle. Antigens and detection antibodies premix in micromixers. Premix simplifies incubation and washing steps and decreases the time for detection with optimization and pretreatment. SA-B system amplifies the signal of IL-6 to overcome the problem of the large content gap between biomarkers. We read CL intensity from the CL image analysis system and analyze the concentrations of biomarkers. Over and above that, WMC-MDP is capable of rapid, robust, accurate and multiplexed analysis of CRP, PCT, and IL-6 in the dynamic battlefield environment for reliably identifying infections.

## Methods

### Materials and instruments

Capture antibodies powders of CRP (CRP-Ab_1_), PCT (PCT-Ab_1_), IL-6 (IL-6-Ab_1_) were obtained from Abcam (UK). CRP, PCT, and IL-6 were purchased from Abcam (UK). Detection antibodies powders of CRP (CRP-Ab_2_), PCT (PCT-Ab_2_) conjugated with horseradish peroxidase (HRP) were purchased from Abcam (UK). Detection antibodies of IL-6 conjugated with Biotin (B-IL-6-Ab_2_) and HRP conjugated with SA (SA-HRP) were obtained from Thermo Fisher Scientific (USA). Bovine serum albumin fraction V (BSA) powders were acquired from Tianjin Kangyuan Biotechnology Co., Ltd. (Tianjin, China). Detection antibodies use 0.05% BSA solution to dilute. Pretreating WMC channels requires a 5% BSA solution. Phosphate Buffered Saline (PBS) tablets and Tween-20 were purchased from Amresco (USA) to prepare 0.01 mol/l PBS solution (pH 7.4) and 0.5% Phosphate Buffered Saline Tween-20 (PBST) solution. The capture antibodies and antigens dilute in PBS solution. Supersensitive chemiluminescent substrate reagent kits were obtained from Beijing Labgic Technology Co., Ltd. (Beijing, China). Polydimethylsiloxane (PDMS) and curing agent (Sylgard 184) were purchased from Dow Corning Inc. (USA) to prepare microfluidic chips. Silicone film (100.0 mm × 100.0 mm × 0.1 mm) used to coat capture antibodies was purchased from Shanghai Shentong Rubber and Plastic Products Co., Ltd. (Shanghai, China). Lasty-R resin from SHINING 3D Technology Co., Ltd. (Yangzhou, China) was the material for 3D printing.

The studies involving human participants were reviewed and approved by the Ethics Committee of Yangzhou University Medical College (YXYLL-2020-89). All subjects provided written informed consents. Testing of clinical samples included samples from 3 healthy individuals and 12 patients. Serum samples were stored at − 80 °C before detection. All experiments were performed in compliance with the guidelines.

Mold manufacturing uses a Lite 600HD 3D printer produced from SHINING 3D Technology Co., Ltd. (Yangzhou, China) DZF-6020A vacuum drying oven, 202-00 T electric thermostatic drying were obtained from LICHEN-BX Co., Ltd. (Shanghai, China). SANHOPTT Co., Ltd. (Shenzhen, China) provided PT-10 s Plasma Cleaner. ISPLab02 Intelligent syringe pump was acquired from DK INFUSETEK Co., Ltd. (Shanghai, China). CL image analysis system was purchased from BIO-OI Co., Ltd. (Guangzhou, China). Characterization of microchannels uses IMM 3000 metallographic microscopy from MEGA Instruments Co., Ltd. (Suzhou, China). L5S UV/VIS Spectrophotometer from INESA Analytical Instrument Co., Ltd. (Shanghai, China) was to test the transmittance.

### Design of the WMC

The sandwich structure design of WMC comprises two parts. Double PDMS layers and a detection layer. It also has additional features containing a valve and two holders. We used SolidWorks® 2016 software to design the three-dimensional model of the WMC.

The channel layer is 68 mm long, 37 mm wide, and 4 mm thick on the top of the sandwich structure. It contains four reservoirs. Each reservoir can accommodate 85 μl reagent. To introduce reagents and equalize the pressure, each reservoir is drilled with a 1 mm vent. The antigens, detection antibodies, and enzymes are mixed together using a wave-shaped micromixer. The mixer consists of five semi-elliptical units of 5.0 mm on the major axis and 3.0 mm on the minor axis. Each unit connection has an end-to-end linkage. There is a valve hole between the micromixer and the reservoir of 8.5 mm in diameter to connect them. At the end of the wave-shaped micromixer, we designed detection channels of three linear channels arranged in parallel with a gap of 3.0 mm. Semicircle channels connect the three linear channels with a negative pressure vent of 1.0 mm. We inserted all vents into flexible tubes for reagents injection and fluid driving. Thus, allowing both positive and negative pressure for fluid driving. All channels are 400 μm in width and depth with a total fluid holding volume of 20 μl. A silicone film of 10.0 mm long, 8.0 mm wide, and 0.1 mm thick is the detection layer coated with capture antibody strips between double PDMS layers. The bottom base layer of WMC has the same size and valve hole as the channel layer mentioned earlier. There is a waste reservoir designed on the base layer for receiving waste reagents.

The design of the outside chip has two holder plates with a thickness of 2.0 mm connected by pins to prop up the chip. Both holder plates have holes to fit the translate-type mechanical (TM) valve to avoid the swinging phenomenon of the TM valve. The upper holder plate consists of another hole to expose the vent to the reservoir. TM valve connects the required reservoirs to the wave-shaped micromixer by setting up channels in different heights. During the movement of the TM valve, the connected reservoir is not dependent on the outlet alignment with the corresponding channel on the TM valve.

### Fabrication and assembly of WMC-MDP

For the fabricating the molds of double PDMS layers and auxiliary parts we used a 3D printer to manufacture [33, 34] (Additional file 1: Fig. S1a). The molds are made out of high-temperature-resistant resin material to avoid mold deformation which occurs due to high temperature during the curing period of the PDMS. Auxiliary parts made of transparent material can observe the fluid motion conveniently. After printing all the parts, they are washed in an ultrasonic cleaner with 99.7% ethanol for 5 min and cured in a UV curing chamber for 15 min. We grind the mold to obtain a surface roughness of Ra = 1.6 μm. It is conducive for higher dimensional accuracy and transmittance. Gloss oil is sprayed on the surface molds’ surface to improve the channel’s shapes. PDMS and curing agents are mixed evenly in a weight ratio of 10:1. A vacuum drying oven is used to debubble the mixture. It is then poured into the molds. The PDMS is cured in the electric thermostatic drying oven at 70 °C for 90 min. After demolding the PDMS, the achievement of the channel layer and base layer are complete. The plasma cleaner breaks the silicon-oxygen bond on the surface and bonds the channel layer and base layer.

We sandwiched the detection layer between two PDMS layers, taking the necessary precautions to ensure that the double PDMS layers covered the whole detection channel region. The capture antibody strips on the detection layer must be perpendicular to the detection channel. A secure bond is ensured by applying adequate pressure to the dual PDMS layers. To accommodate the bonded PDMS layers, two holding plates are built using pins. To facilitate sample addition and fluid drive in the WMC, a TM valve is used in the valve holes. Flexible tubes of appropriate length are used in all the vents to connect with the pump. Finally, the assembly of WMC-MDP is achieved (Additional file 1: Fig. S1b).

### Pretreatment of WMC-MDP

All three channels of our microfluidic chip design are capable of coating the silicone sheet with capture antibodies. The channels are 400 μm in width and depth, also are parallel and spaced at 3 mm intervals. The silicone film is cut to the design’s dimensions and placed along its length (Additional file 1: Fig. S2a). CRP-Ab_1_, PCT-Ab_1_, IL-6-Ab_1_ are injected into the channels respectively, followed by a 20-min period of quiescence (Additional file 1: Fig. S2b). After that, PBST buffer is injected three times into each channel to eliminate uncoated capture antibodies (Additional file 1: Fig. S2c). Finally, the chip is removed and the detecting layer coated with captured antibodies (Additional file 1: Fig. S2d).

We then added 50 μl of 5% BSA solution to any reservoir by pressing the TM valve to the appropriate height on the completed WMC-MDP. Positive pressure is used to force the reagents through the reservoir vents, while negative pressure is used to drive them through the negative pressure vent. After completely filling all channels with BSA solution, WMC-MDP is left inactive for 30 min to minimize non-specific protein adsorption. Additionally, the addition of the BSA solution serves as a quality control measure. If liquid spills during the adding procedure, the chip’s quality is compromised (Additional file 1: Fig. S3).

## Results

### Principle

The basic steps of WMC-MDP in detecting CRP, PCT, and IL-6 include preprocessing, premixing, and signal readout. Pretreatment involves coating the detection layer with captured antibody strips and modifying the surface of the channels. The purpose of premixing is to mix the antigens with antibodies for detection before conducting an immune reaction. Pre-mixing antigens and antibodies in a micromixer can eliminate the need for incubation and washing, saving half of the time of detection. The reaction of HRP with substrate of Luminol-H_2_O_2_ deconstructs H_2_O_2_ into an oxygen-free radical, which is catalyzed by HRP and oxidizes the luminol to produce CL signals. Concentration of CRP and PCT are high in serum and can be detected by HRP-conjugated antibodies directly. However, due to the low concentration of IL-6 in the serum, we need to introduce a signal amplification system. We used the SA-B system to detect IL-6 by conjugating biotin with antibodies and streptavidin with HRP. The preparations of the two conjugates are mature and commercial processes. An automatic system reads the CL signals and analyzes the CL image.

### Characterization

**Fig. 1:**
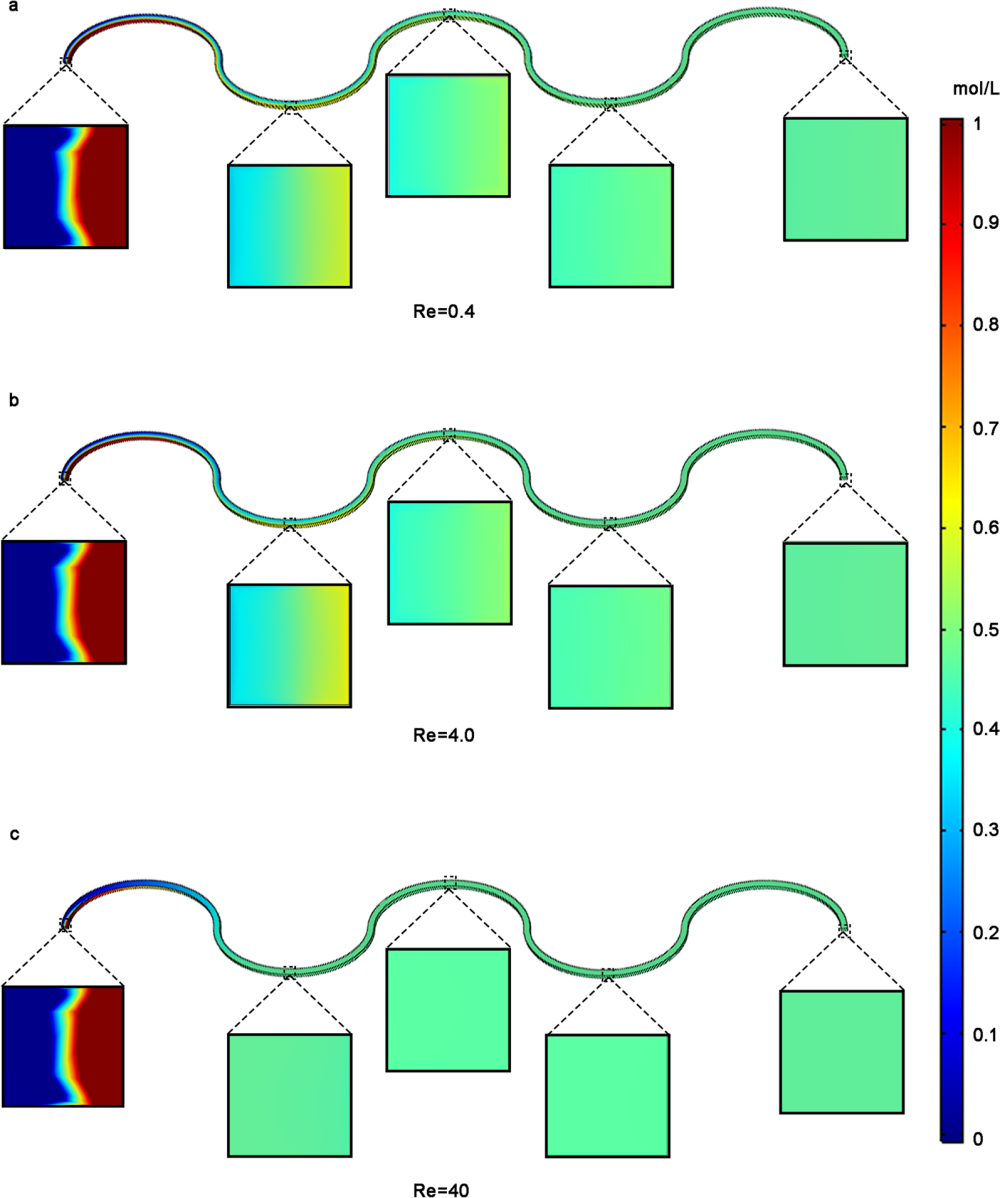
Simulation of the mixing effect of wave-shaped micromixer under different Re using COMSOL software. **a** When the Re was 0.4, the mixing efficiency at the outlet was 0.99606. **b** Again with the Re of 4 the mixing efficiency at the outlet was 0.99874. **c** Under the Re of 40 the mixing efficiency at the outlet observed was 0.99999. Re Reynolds number

The micromixer is a reliable structure integrated with microfluidic chip [35–37]. Effectiveness of the micromixer relies on evenly mixing of proteins in the samples. It is an essential prerequisite for WMC-MDP to reduce the time of detection by premixing. We used COMSOL Multiphysics 5.6 software to simulate the condition of mixing in the three-dimensional model of the wave-shaped micromixer and explore the effect of mixing reagents in the wave-shaped micromixer. The judgement does not rely on motion changes of an individual protein, so there is no need to consider biomechanical aspects. Driven by the pump, the fluid in WMC-MDP moves at Reynolds numbers (Re) of 0.4 (Fig. 1a), 4 (Fig. 1b), and 40 (Fig. 1c). To investigate whether two kinds of liquid could transform into a homogeneous liquid, we mixed liquid with the concentration of 0 mol/l and 1 mol/l in the wave-shaped micromixer. To quantify the mixing impact, we evaluate the mixing efficiency using the following formula.

$$\sigma = 1 - \sqrt {\frac{{\int_{\Gamma } {\left( {c - \overline{c} } \right)^{2} } }}{{A \times \overline{c} \times 2}}}$$

where $c$ is the concentration of the liquid, $\overline{c}$ is the average concentration, $\Gamma$ is the concentration we measured from the outlet plane, $A$ is the area of the outlet plane.

When the Re is 0.4, 4, 40, the resulting mixing efficiency is 0.99606, 0.99874, and 0.99999, respectively. WMC-MDP is capable of effective premixes at a wide range of the Re, and it can satisfy the requirements from manual operations to instrumental automation.

**Fig. 2:**
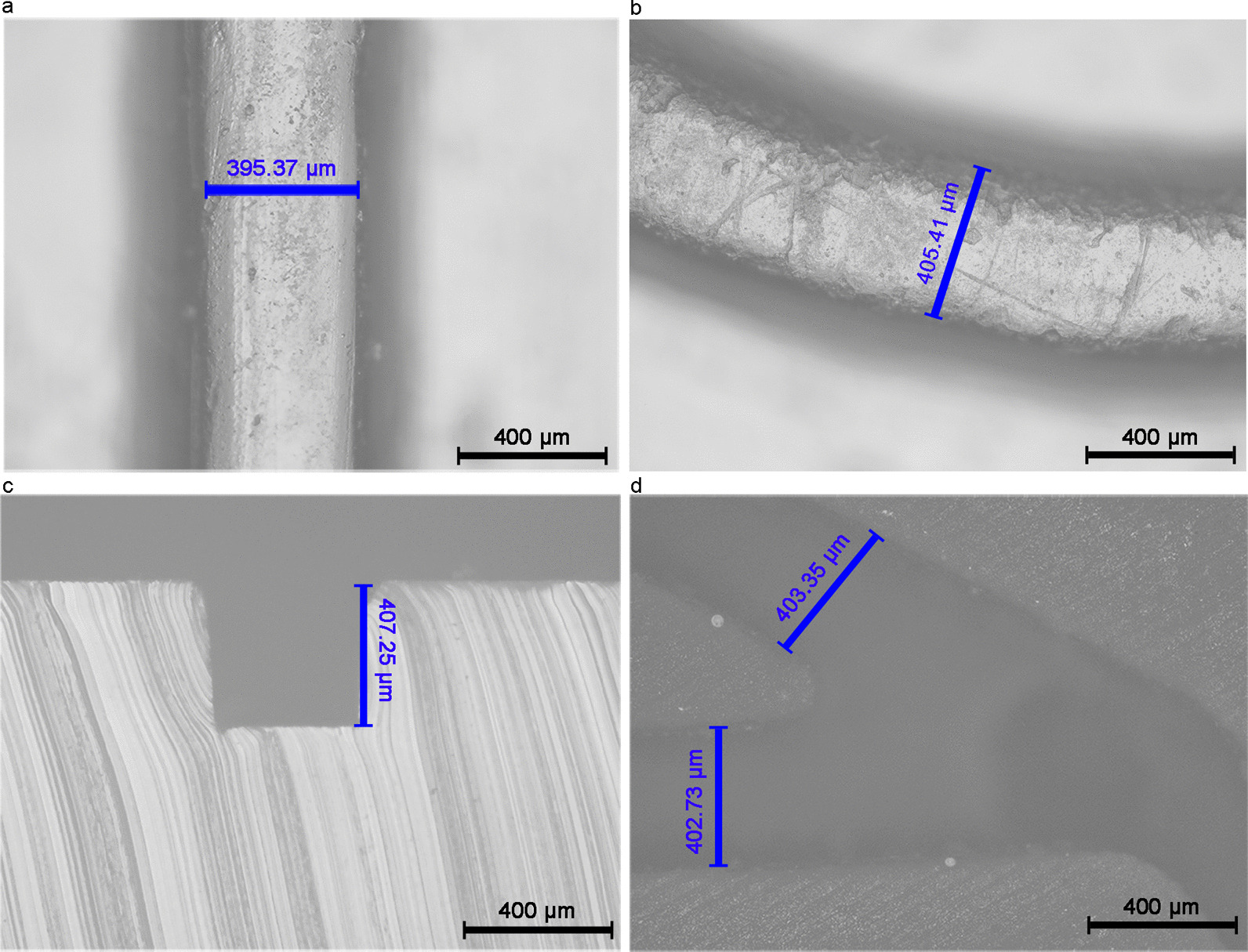
Dimensions of channels in WMC components. **a** WMC microchannels longitudinal part is investigated with the designed width of 400 μm and characterized, and the actual width is 395.37 μm with a dimensional deviation of 1.16%. **b** Curved channel of WMC is also measured according to the width design of 400 μm. But the actual width is 405.41 μm, and the dimensional deviation is 1.35%. **c** Depth of the channel in WMC has a designed height of 400 μm. But in reality, the height is 407.25 μm with a dimensional deviation of 1.81%. **d** Compared with the designed 400 μm width of channels in the TM valve, it has 402.73 μm and 403.35 μm. Here the mean dimensional deviation is 0.61%. WMC wave-shaped microfluidic chip, TM valve translate-type mechanical valve

Accurate size is the basis for ensuring normal operation of the micromixer, smooth fluid movement, and sensitivity of detection. 3D-printed mold is an emerging technology in the manufacture of microfluidic chips [38]. To verify the reliability of manufacturing WMC mold when using 3D printing technology, we used microscopy to measure the dimensions of the channels in the WMC. The dimensional deviation of the straight and curved channels in the channel layer (Fig. 2a–c), channel in the TM valve (Fig. 2d) is within 2%. It proves that WMC manufactured using 3D printing has extremely high manufacturing accuracy and can meet the requirements of mass production.

In addition to dimensional accuracy, the transmittance is another criterion to evaluate manufacturing of chips. To observe the movement of fluid in the chip and detect the CL intensity, we examined the transmittance of WMC in the visible region (380–720 nm) (Additional file 1: Fig. S4). The results showed that the average transmittance of WMC in the visible region is 85.97%. At the optimal condition for detecting the CL intensity (425 nm) [39], the transmittance of WMC is 86.04%. Compared with the 95% transmittance of PDMS, the light transmittance of WMC does not decrease significantly and meets the requirements of observation and detection.

### Optimization of coating capture antibodies

We coated antibodies with different concentrations to optimize concentrations of antibodies for capturing CRP, PCT, and IL-6 onto the detection layer. We investigated CRP-Ab_1_ and PCT-Ab_1_ of 10, 20, 40, 60, 80 μg/ml (Additional file 1: Fig. S5a and b), IL-6-Ab_1_ of 20, 40, 60, 80, 120 μg/ml for coating (Additional file 1: Fig. S5c). The concentrations of CRP-Ab_2_, PCT-Ab_2_, and B-IL-6-Ab_2_ were all 75 μg/ml. The concentration of SA-HRP was 4 μg/ml. The other experimental conditions are consistent. Initially, CL intensity is positively correlated with the concentration of capture antibodies. When the concentrations of capture antibodies rise to a certain extent, increase of CL intensity becomes inconspicuous. The CL intensity even decreases when the concentrations of capture antibodies are at high levels. The optimal conditions for capturing CRP-Ab_1_, PCT-Ab_1_, IL-6-Ab_1_ are 40, 60, and 80 μg/ml. We used the optimal concentrations for coating.

### Optimization of detection antibodies

Detection antibodies with the concentrations of 6.25, 12.5, 25, 50, and 75 μg/ml are used to investigate the optimal conditions for detecting CRP (Additional file 1: Fig. S6a), while detection antibodies with the concentrations of 12.5, 25, 50, 75, 100 μg/ml are used for detecting PCT and B-IL-6 (Additional file 1: Fig. S6b and c). The intensity of CL is enhanced by increasing the concentration of detection antibodies until it reaches a plateau. Therefore, we chose 25 μg/ml of CRP-Ab_2_, 50 μg/ml of PCT-Ab_2,_ and B-IL-6-Ab_2_ as the optimal concentration.

### Detecting process

**Fig. 3:**
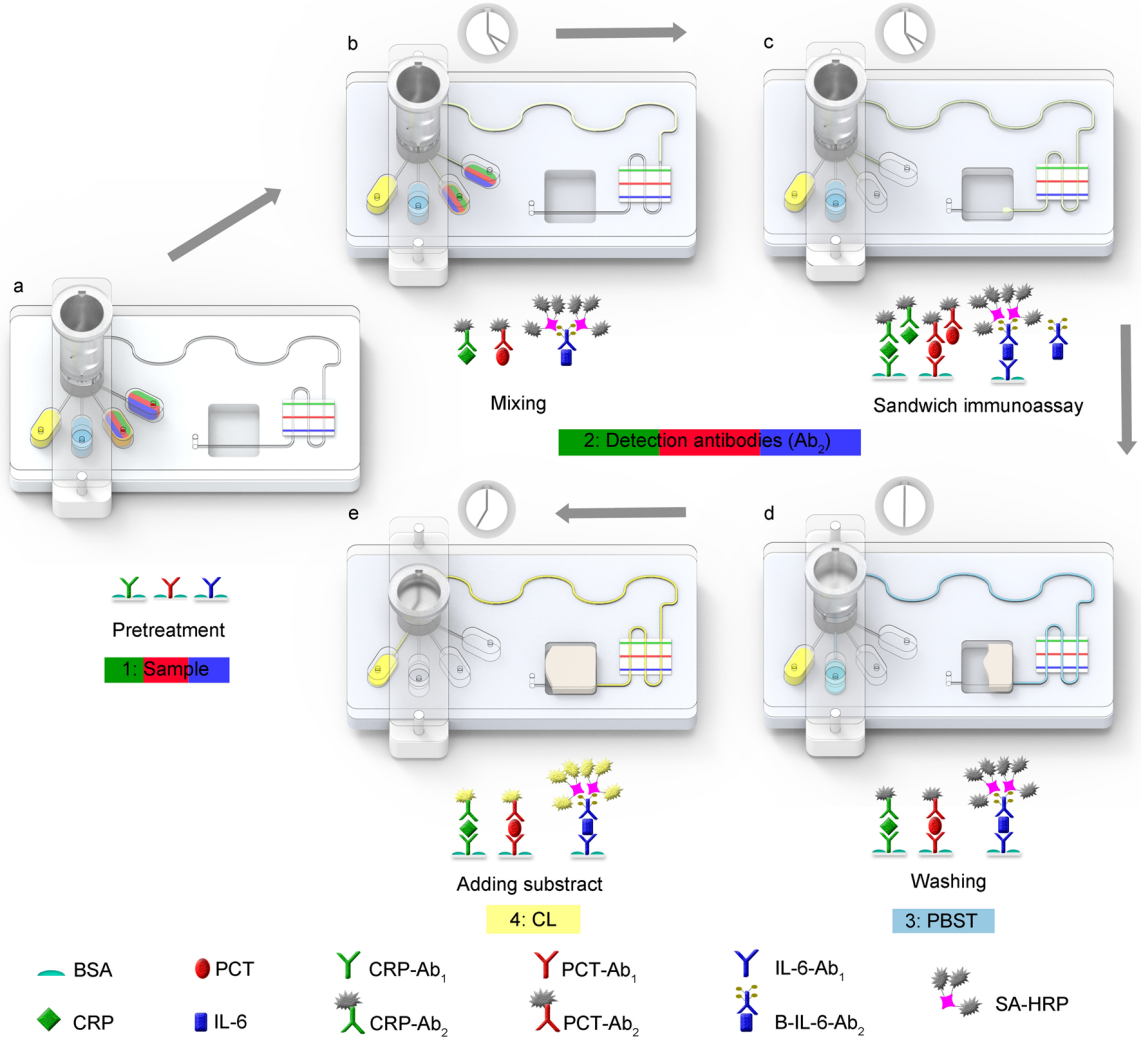
Schematic diagram for multiplex detection of CRP, PCT, and IL-6 using WMC-MDP. **a** Prior to detection, pretreatment includes coating capture antibody strips, surface preparation of channels, and the addition of reagents. **b** Premixing samples with detection antibodies in wave-shaped micromixer. **c** Sandwich immunoassay occurs in the detected channels and forms structures of Ab_1_-CRP-Ab_2_-HRP, Ab_1_-PCT-Ab_2_-HRP, and Ab_1_-IL-6-Ab_2_-B-SA-HRP. **d** Removing unreacted reagents by washing channels. **e** Adding CL substrate into the channels to produce CL intensity. CRP c-reactive protein, PCT procalcitonin, IL-6 interleukin-6, WMC-MDP wave-shaped microfluidic chip assisted multiplexed detection platform, PBST phosphate buffered saline tween-20, CL chemiluminescence, BSA bovine serum albumin, CRP-Ab_1_ capture antibodies of c-reactive protein, PCT-Ab_1_ capture antibodies of procalcitonin, IL-6-Ab_1_ capture antibodies of interleukin-6, CRP-Ab_2_ detection antibodies of c-reactive protein, PCT-Ab_2_ detection antibodies of procalcitonin, B-IL-6-Ab_2_ detection antibodies of interleukin-6 conjugated with biotin, HRP horseradish peroxidase, SA-HRP horseradish peroxidase conjugated with streptavidin

**Fig. 4:**
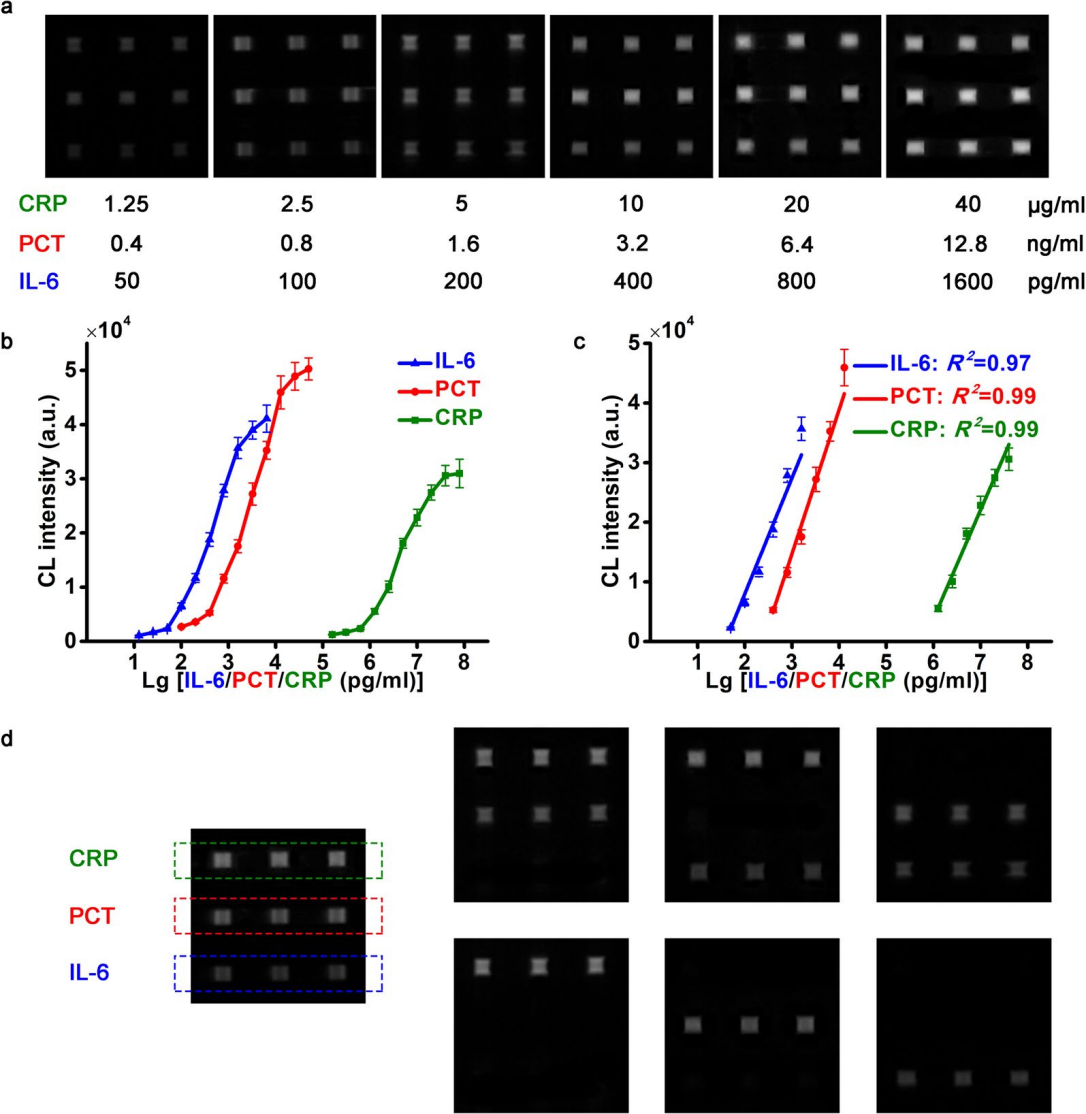
CL intensity is obtained from multiplex detection of CRP, PCT, and IL-6 using WMC-MDP to construct detection range, linear range, and selectivity. **a** CL pictures of CRP, PCT, and IL-6 in the linear range at various concentrations. The scan shows CRP in the upper left corner, PCT in the middle, and IL-6 in the lower corner. **b** Detection range of CRP, PCT, and IL-6. **c** Liner range of CRP, PCT, and IL-6. **d** CL images of CRP, PCT, and IL-6 in different combinations. CL chemiluminescence, CRP C-reactive protein, PCT procalcitonin, IL-6 interleukin-6, WMC-MDP wave-shaped microfluidic chip assisted multiplexed detection platform

We used a negative pressure peristaltic pump to drive the fluid. First, we connect the pump to the negative pressure vent. Then 30 μl of the sample, 40 μl of the mixed solution (detection antibody and SA-HRP), 70 μl of PBST and 35 μl of the substrate are added into the four reservoirs, respectively (Fig. 3a). Steps for detection contain: (1) Pressing the TM valve to the appropriate height to ensure the sample and detection antibodies with SA-HRP solution can enter the wave-shaped micromixer, (2) Pumping the mixed solution into the reaction channel, (3) Positioning the TM valve by pressing it to the next height to pump PBST solutions into the detection channel to remove unreacted reagents (Fig. 3d), (4) Pumping CL substrate into detection channel to react with HRP after pressing the TM valve to the last height. In the micromixer, the reagents can be evenly mixed and initially reacted (Fig. 3b). In the second step, solutions are incubated for 20 min to generate an immunoreaction, forming a double-antibodies sandwich structure. (Fig. 3c). Finally, strips of capture antibodies and detection channels cross to form a 3 × 3 detection spots. We obtained the CL intensity by the CL image analysis system and used GEL-PRO Analyzer software for subsequent analysis (Fig. 3e). The entire process takes 22 min.

### Detecting performance

Linear range, limit of detection (LOD), and selectivity can evaluate the detection performance of WMC-MDP. Under the optimal conditions, we detected CRP with the range of 0.16–80 μg/ml, PCT with the range of 0.1–51.2 ng/ml, and IL-6 with the range of 12.5–6400 pg/ml (Fig. 4a, b). The linear ranges are 1.25–40 μg/ml for CRP ($Y = - 105726.34 + 18258.99X$; $R^{2} = 0.99$), 0.4–12.8 ng/mL for PCT ($Y = - 57834.93 + 24184.26X$; $R^{2} = 0.99$). 50–1600 pg/ml for IL-6 ($Y = - 30857.38 + 19426.37X$; $R^{2} = 0.97$) (Fig. 4c). The linear ranges cover the cut-off values of the three biomarkers. $LOD = 3S/M$, while *S* is the standard deviation of the blank samples, and *M* is the slope of the linear curve. The LODs of CRP, PCT and IL-6 are 0.54 μg/ml, 0.11 ng/ml and 16.25 pg/ml, respectively. Since the LODs are far lower than the cut-off values, the sensitivity of the three biomarkers can meet the needs for POCT.

No cross-reactions are the premise to realize multiplexed detection of the three biomarkers. We detected all the combinations of CRP, PCT, and IL-6 to observe if they would make a difference (Fig. 4d). The detection process is consistent except for the different combinations of samples in the reservoir. The concentrations of CRP, PCT and IL-6 are 10 μg/ml, 0.8 ng/ml and 100 pg/ml. The results show that the CL signals can only appear when the corresponding captured antibodies are coated. Different combinations of biomarkers do not affect the CL intensity of the detection spots. It indicates that cross-reactions were not generated, and WMC-MDP is capable of multiplexed detection of infectious biomarkers.

### Reproducibility

Reproducibility is also an important index to evaluate the detection capability of the WMC-MDP. We used ten pieces of WMC produced from the same batch to detect the same sample. The concentrations of CRP, PCT, and IL-6 in the samples are 10 μg/ml, 0.8 ng/ml, and 100 pg/ml. The results show that the coefficients of variation (CV) for CRP, PCT, and IL-6 are 5.24% (Additional file 1: Fig S7a), 5.02% and 6.12%, respectively (Additional file 1: Fig S7b and c). All the CV are less than 10%, which indicates that the reproducibility of WMC-MDP is compatible for POCT application. The precise fabrication of the WMC and optimization of the experimental conditions ensure optimum reproducibility.

### Storage stability

To satisfy the demands of POCT and commercialization, WMC-MDP needs to have excellent storage stability. We investigated the detection capability of WMC-MDP after storing for 1–7 d at a temperature of 4 °C and a pressure of 101 kPa. The concentrations of CRP, PCT, and IL-6 to be detected are 10 μg/ml, 0.8 ng/ml, and 100 pg/ml respectively. The signals display no distinct decrease in the WMC-MDP for detecting the same concentrations of samples. The CV of CRP, PCT, and IL-6 are 6.33%, 5.77%, and 6.64%, respectively, less than 15% (Additional file 1: Fig S8). It indicates WMC-MDP can stably store the reagents.

### Clinical sample testing

**Fig. 5:**
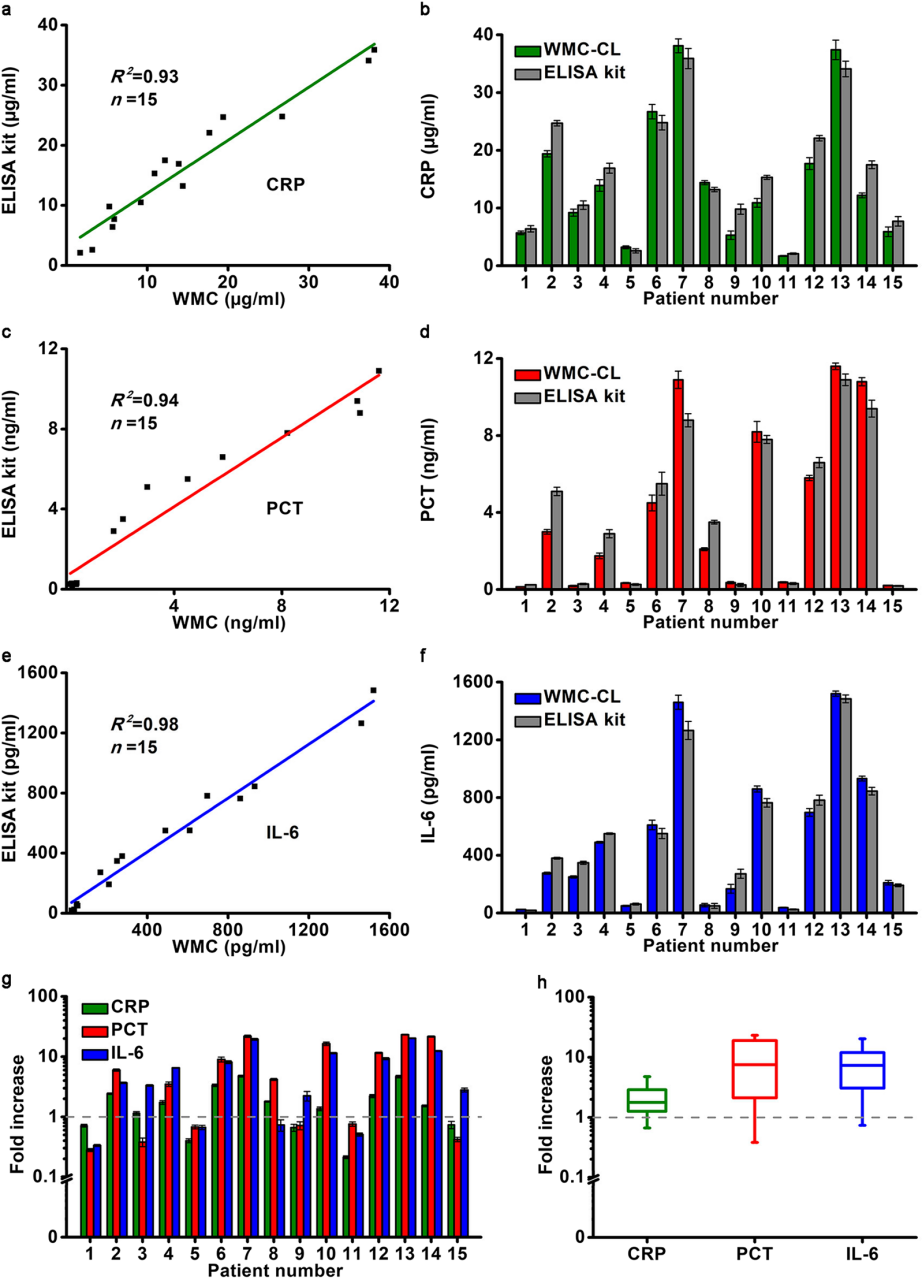
Comparing and analyzing detection data of CRP, PCT, and IL-6 in clinical samples between WMC-MDP and commercial ELISA kits. **a** and **b** Comparison of WMC-MDP and ELISA Kits for CRP detection. **c** and **d** Comparison of WMC and ELISA Kits for PCT detection. **e** and **f** Comparison of WMC-MDP and ELISA Kits for IL-6 detection. **g** Analysis of CRP, PCT, IL-6 in clinical samples detected by WMC-MDP, without logarithmic transformation at 0. **h** Box-and-whisker plot of CRP, PCT, and IL-6 in 12 patients with infection. CRP C-reactive protein, PCT procalcitonin, IL-6 interleukin-6, WMC wave-shaped microfluidic chip, WMC-MDP wave-shaped microfluidic chip assisted multiplexed detection platform, WMC-CL wave-shaped microfluidic chip using chemiluminescence, without logarithmic transformation at 0

We detected 15 serum samples to verify the capability of WMC-MDP in clinical diagnosis and compared the results with commercial ELISA kits. The justification highlights the potential of WMC-MDP in practical applications. The results of WMC-MDP are highly consistent with commercialized ELISA kits (CRP, $R^{2} = 0.93$; PCT, $R^{2} = 0.94$; IL-6, $R^{2} = 0.98$) (Fig. 5a–f). Results of samples from 3 healthy volunteers and 12 sick volunteers are all consistent with clinical diagnostic results.

The reasonable preset and uniform cut-off values facilitate the analysis of clinical samples. We used cut-off values of 8 μg/ml, 0.5 ng/ml, and 75 pg/ml for CRP, PCT, and IL-6, respectively. For visualizing the changes of biomarkers, we used the fold increase of them compared with the cut-off value to show the contents of biomarkers (Fig. 5g). From the perspective of patients No. 9 and No. 15, only the level of IL-6 in the patients’ blood is higher than the cut-off value. It may be due to the early stage of the infection. Levels of CRP in blood begin to rise after 6–8 h of infection, and achieve to peak after 24–48 h. Levels of PCT rise within 2–4 h and reach a plateau within 12–48 h, while levels of IL-6 rise quickly, but with a half-life of only 1 h. It indicates that the samples should be collected soon after the patient was infected for detection of IL-6. Bacteria, viruses and other pathogens can cause infections. PCT is only sensitive to bacterial infection, while CRP is not sensitive to this pathogen. Levels of IL-6 are high in patient No. 3, while levels of CRP levels are slightly elevated, and levels of PCT are normal. It may be caused by other pathogens, rather than bacterium. Medication use may also result in a rapid decline in PCT, which needs further diagnosis according to the patient’s medical history. Patient No.8 aroused our interest in the high levels of CRP and PCT in blood and normal levels of IL-6. It may be because the patient’s infection was not severe, and IL-6 levels have declined due to its short half-life, while PCT and CRP have not. The box-and-whisker plot shows infected patients can also have a fold increase of biomarkers levels less than one (Fig. 5h). It means even if a person is infected, concentrations of certain biomarkers may be within the normal range. According to the above case analysis, it is difficult to diagnose infection with an individual biomarker accurately. Multiplexed detections of CRP, PCT, and IL-6 can fill in the blind area of single biomarker analysis and accurately diagnose the infection.

## Discussion

The overall performance of WMC-MDP is satisfactory when compared to existing mature technologies. It is due to the advanced design and precise manufacture of the modules including processing channels, micromixers, reaction layer materials, and reagent storage.

Combining 3D printing with microfluidic technologies opens up a new way for mass production of chips. The width of channels in the WMC-MDP is 400 μm, showing better manufacturing accuracy than injection molding. However, the manufacturing error increases significantly when the channel width is less than 50 μm. In addition, PDMS is difficult to cure in molds produced using photocuring 3D printing technology. Physical isolation or surface modification can solve the problem but it complicates the manufacturing process. New materials and technological improvements may provide promising alternatives for fabricating microfluidic chips based on 3D printing. Micromixers have been widely used in microfluidic chips. Topology-based micromixers are being developed to mix fluids in shorter channels. However, the complex structure makes it difficult to manufacture. In addition, it can hardly meet the requirements of fluid mixing at wide range of Re. Reasonable design of WMC-MDP makes the wave-shaped micromixer occupy minimal space. It allows the WMC-MDP to be portable while adapting to complex battlefield environments.

Although we shorten the incubation time, the sensitivity is not affected, due to the good adsorption capacity of the reaction layer. Instead of the commonly used aluminum film, we chose silicon film as the material of reaction layer. Compared with the aluminum film, the adsorption capacity of the silicon film on protein is greatly improved. With strong adsorption and good plasticity, silicon film is capable of amplifying signals of detection. For microfluidic chips, storage of reagents has been one of the most challenging issues. Even though WMC-MDP can keep the reagent at 4 °C for more than 7 d, it is still a long way to make the method suitable for market use It may be alleviated by using new freeze-drying techniques or improving the structure of reservoirs.

We compared the capability of multiplexed detection, as well as operation, time, cost and LOD of detection based on WMC-MDP with conventional detection methods (Table 1). Compared with ELISA kits, fluorescence immunoassay, and ECL immunoassay, WMC-MDP demonstrates better performances in regard to operation, time and cost. Although the LOD of IL-6 is higher than fluorescence immunoassay and ECL immunoassay, it still meets the requirements of diagnosing infectious diseases. In addition, WMC-MDP can realize the multiplex detection of three biomarkers. It has practical significance in clinical application. The performance of WMC-MDP in clinical sample analysis also validated its application value in rapid diagnosis and classification of infection. The rapidity, low cost, minimal manual intervention and capability of multiplexed detection make WMC-MDP a strong candidate for military-oriented POCT.

**Table 1:** Comparison of WMC-MDP detection capabilities

Detection method	WMC-MDP	CL [40]	Colorimetric	Fluorescence [41]	ECL [42]
Multiplex detecting	Y	Y	N	Y	N
Detection time (min)	22	70	90	33	> 240
Cost	Low	Medium	High	High	Medium
Performance of operations	Simple	Simple	Complicated	Simple	Complicated
LOD					
CRP (μg/ml)	0.54	1.87	1.19	5.53	/
PCT (ng/ml)	0.11	0.17	0.15	/	/
IL-6 (pg/ml)	16.25	49.75	12.5	4.41	1

*WMC-MDP* wave-shaped microfluidic chip assisted multiplexed detection platform, *CL* chemiluminescence, *ECL* electro-chemiluminescence, *Y* yes, *N* no, *LOD* limit of detection, *CRP* c-reactive protein, *PCT* procalcitonin, *IL-6* interleukin-6

## Conclusion

In conclusion, to investigate infection in a military scenario, we develop a WMC-MDP for multiplexed detections of CRP, PCT, and IL-6. Premixing of antigens and antibodies for detection simplifies steps of reaction and shortens the time for detection. A dependable process of manufacturing and optimization of conditions for reaction improve the sensitivity and specificity, while reducing the cost. Pretreatment simplifies the process of operations. Advantages of rapidity, cost-effectiveness and user-friendliness make WMC-MDP a competitive alternative to traditional methods. WMC-MDP provides a feasible method for the detection of other pathogens. WMC-MDP is a prospective tool for rapid identification of infections and shows the potential to be developed to militarized POCT devices.

## Supplementary Information

**Additional file 1: Fig. S1.** A real wave-shaped microfluidic chip (WMC) assisted multiplexed detection platform (WMC-MDP). **Fig. S2.** Process of coating capture antibodies strips on detection layer. **Fig. S3.** The red ink flows in the channel. No liquid leakage indicates that the chip is qualified. **Fig. S4.** Transmittance of WMC is in the range of 380-720 nm. **Fig. S5.** Optimization of capture antibodies in WMC-MDP. **Fig. S6.** Optimization of detection antibodies in WMC-MDP. **Fig. S7.** Reproducibility of CRP, PCT, and IL-6. **Fig. S8.** Storage stability of CRP, PCT, and IL-6.

## Data Availability

The data and materials used in the current study are all available from the corresponding author upon reasonable request.

## Abbreviations

B-IL-6-Ab_2_: Detection antibodies of IL-6 conjugated with biotin
BSA: Bovine serum albumin
CL: Chemiluminescence
COVID-19: Corona Virus Disease 2019
CRP: C-reactive protein
CRP-Ab_1_: Capture antibodies of CRP
CRP-Ab_2_: Detection antibodies of CRP
CV: Coefficients of variation
ECL: Electro-chemiluminescence
H1N1: Hemagglutinin 1 Neuraminidase 1
HRP: Horseradish peroxidase
IL-6: Interleukin-6
IL-6-Ab_1_: Capture antibodies of IL-6
LOD: Limit of detection
PBS: Phosphate buffered saline
PBST: Phosphate buffered saline tween-20
PCT: Procalcitonin
PCT -Ab_1_: Capture antibodies of PCT
PCT -Ab_2_: Detection antibodies of PCT
PDMS: Polydimethylsiloxane
POC: Point-of-care
POCT: Point-of-care testing
Re: Reynolds number
SA-B: Streptavidin–biotin
SA-HRP: HRP conjugated with SA
TM valve: Translate-type mechanical valve
WMC: Wave-shaped microfluidic chip
WMC-MDP: Wave-shaped microfluidic chip assisted multiplexed detection platform
