# Supplementary material for: Wave-shaped microfluidic chip assisted point-of-care testing for accurate and rapid diagnosis of infections
Source: Mil Med Res. 2022 Feb 11;9:8. doi: 10.1186/s40779-022-00368-1 (PMC8831027; doi:10.1186/s40779-022-00368-1)
Supplement: Supplementary file 1 — Additional file 1: Fig. S1. A real wave-shaped microfluidic chip (WMC) assisted multiplexed detection platform (WMC-MDP). Fig. S2. Process of coating capture antibodies strips on detection layer. Fig. S3. The red ink flows in the channel. No liquid leakage indicates that the chip is qualified. Fig. S4. Transmittance of WMC is in the range of 380-720 nm. Fig. S5. Optimization of capture antibodies in WMC-MDP. Fig. S6. Optimization of detection antibodies in WMC-MDP. Fig. S7. Reproducibility of CRP, PCT, and IL-6. Fig. S8. Storage stability of CRP, PCT, and IL-6. [file 40779_2022_368_MOESM1_ESM.pdf]

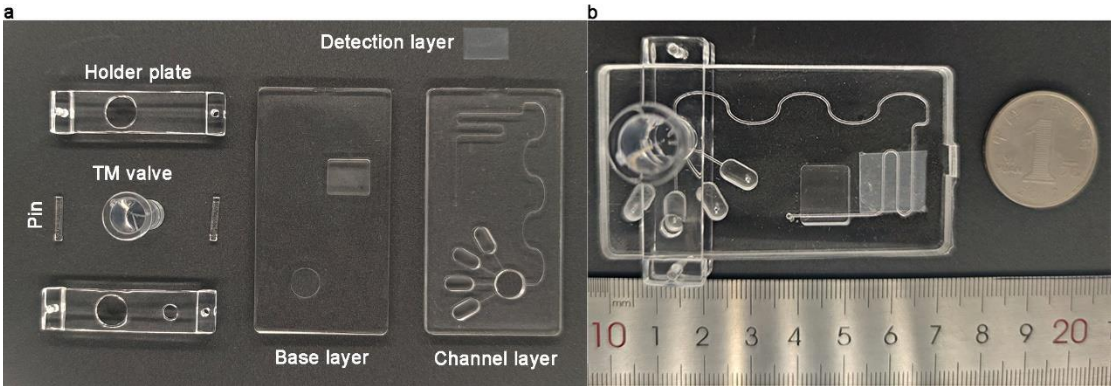

Fig. S1 A real wave-shaped microfluidic chip (WMC) assisted multiplexed detection platform (WMC-MDP). **a** WMC-MDP components include channel layer, detection layer, base layer, holder plate, TM valve, and pins. **b** Image of the assembled WMC-MDP. The detection layer is placed between the channel and base layers to form the sandwich structure, and the TM valve is inserted through the sandwich structure to fit the additional features. WMC wave-shaped microfluidic chip, WMC-MDP wave-shaped microfluidic chip assisted multiplexed detection platform, TM valve translate-type mechanical valve

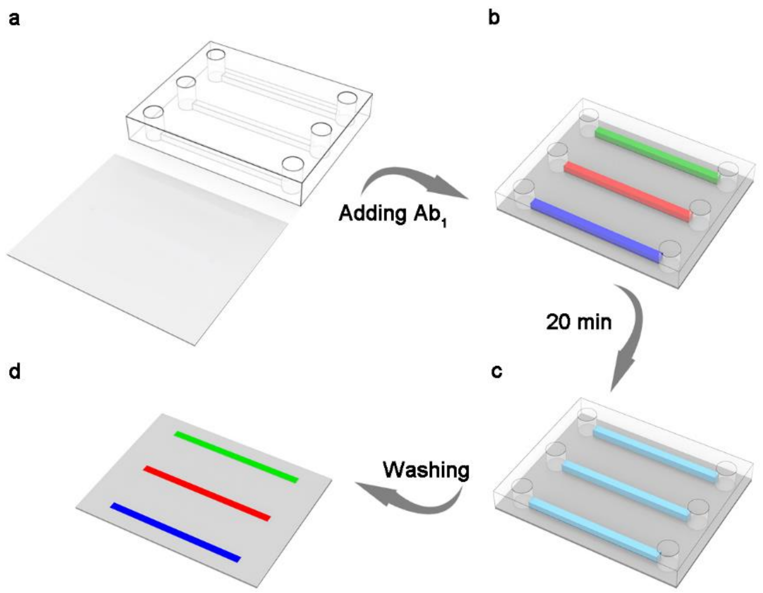

Fig. S2 Process of coating capture antibodies strips on detection layer. **a** Preparing a chip to coat capture antibodies strips and cutting the silicone film according to the designed size. **b** Placing the chip on the silicone film and injecting CRP-Ab_1, PCT-Ab_1, IL-6-Ab_1 into the channels. **c** After incubation for 20 min, injecting PBST buffer into channels to wash three times. **d** Removing the chip and obtaining the detection layer coated with capture antibodies strips. CRP-Ab_1 capture antibodies of c-reactive protein, PCT-Ab_1 capture antibodies of procalcitonin, IL-6-Ab_1 capture antibodies of interleukin-6, PBST phosphate buffered saline tween-20

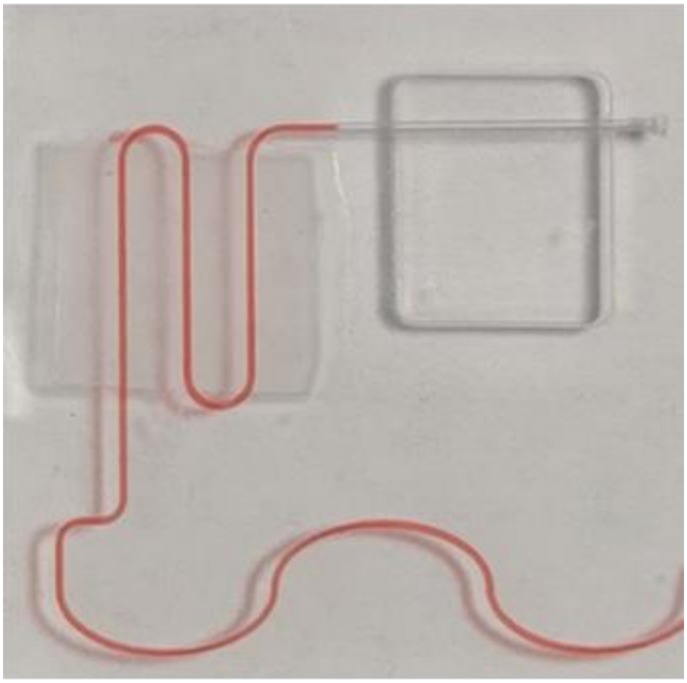

Fig. S3 The red ink flows in the channel. No liquid leakage indicates that the chip is qualified

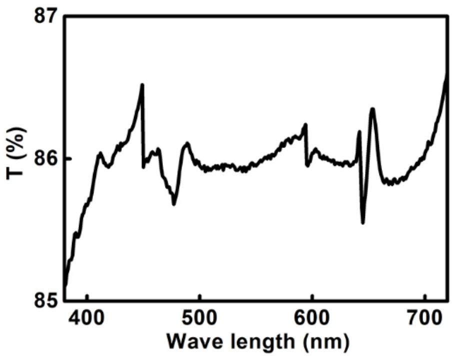

Fig. S4 Transmittance of WMC is in the range of 380-720 nm. The average transmittance is 85.97%. The transmittance at 425 nm is 86.04%. T transmittance, WMC wave-shaped microfluidic chip

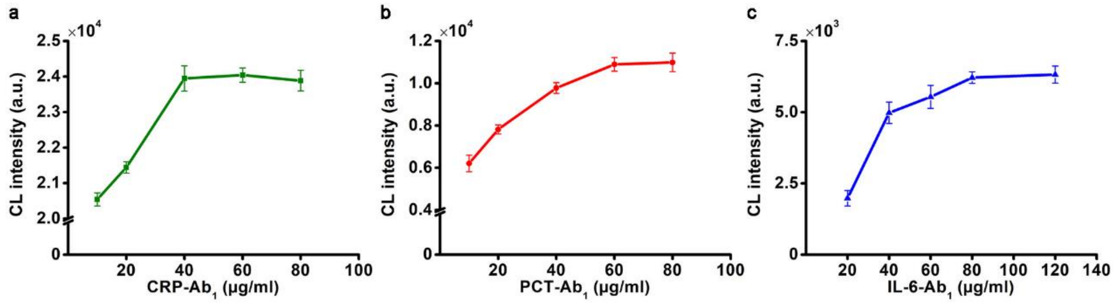

Fig. S5 Optimization of capture antibodies in WMC-MDP. **a** CL intensity for detecting 10 μg/ml of CRP with 10, 20, 40, 60, 80 μg/ml of CRP-Ab$_1$ and 75 μg/ml of CRP-Ab$_2$. **b** CL intensity for detecting 0.8 ng/ml of PCT with 10, 20, 40, 60, 80 μg/ml of PCT-Ab$_1$ and 75 μg/ml of PCT-Ab$_2$. **c** CL intensity for detecting 100 pg/ml of IL-6 with 20, 40, 60, 80,120 μg/ml of IL-6-Ab$_1$, 75 μg/ml of B-IL-6-Ab$_2$ and 4 μg/ml of SA-HRP. WMC-MDP wave-shaped microfluidic chip assisted multiplexed detection platform, CL chemiluminescence, CRP c-reactive protein, CRP-Ab$_1$ capture antibodies of c-reactive protein, CRP-Ab$_2$ detection antibodies of c-reactive protein, PCT procalcitonin, PCT-Ab$_1$ capture antibodies of procalcitonin, PCT-Ab$_2$ detection antibodies of procalcitonin, IL-6 interleukin-6, IL-6-Ab$_1$ capture antibodies of interleukin-6, B-IL-6-Ab$_2$ detection antibodies of interleukin-6 conjugated with biotin, SA-HRP horseradish peroxidase conjugated with streptavidin

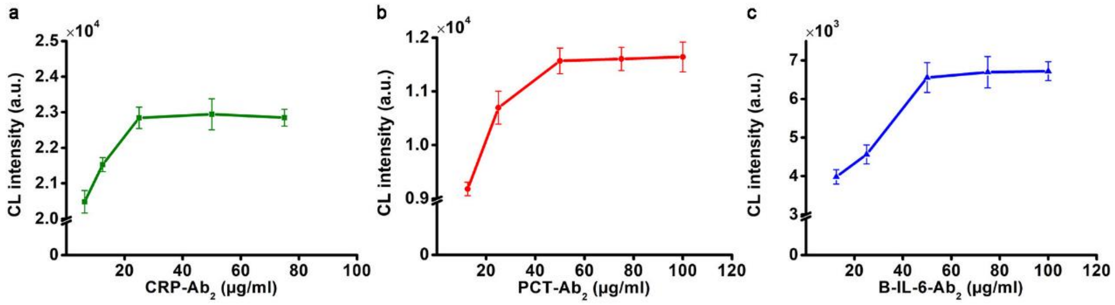

Fig. S6 Optimization of detection antibodies in WMC-MDP. **a**. CL intensity for detecting 10 μg/ml of CRP with 6.25, 12.5, 25, 50, 75 μg/ml of CRP-Ab_2 and 40 μg/ml of CRP-Ab_1. **b** CL intensity for detecting 0.8 ng/ml of PCT with 12.5, 25, 50, 75, 100 μg/ml of PCT-Ab_2 and 60 μg/ml of PCT-Ab_1. **c** CL intensity for detecting 100 pg/ml of IL-6 with 12.5, 25, 50, 75, 100 μg/ml of IL-6-Ab_2, 80 μg/ml of B-IL-6-Ab_1 and 4 μg/ml of SA-HRP. WMC-MDP wave-shaped microfluidic chip assisted multiplexed detection platform, CL chemiluminescence, CRP c-reactive protein, CRP-Ab_2 detection antibodies of c-reactive protein, CRP-Ab_1 capture antibodies of c-reactive protein, PCT procalcitonin, PCT-Ab_2 detection antibodies of procalcitonin, PCT-Ab_1 capture antibodies of procalcitonin, IL-6 interleukin-6, B-IL-6-Ab_2 detection antibodies of interleukin-6 conjugated with biotin, IL-6-Ab_1 capture antibodies of interleukin-6, SA-HRP horseradish peroxidase conjugated with streptavidin

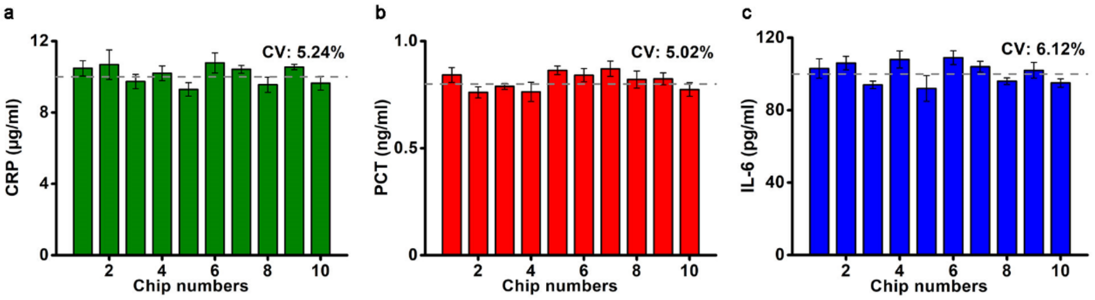

Fig. S7 Reproducibility of CRP, PCT, and IL-6. **a** Results of detecting 10 μg/ml CRP repeatedly in ten chips. **b** Results of detecting 0.8 ng/ml PCT repeatedly in ten chips. **c** Results of detecting 100 pg/ml IL-6 repeatedly in ten chips. CRP c-reactive protein, PCT procalcitonin, IL-6 interleukin-6, CV coefficients of variation

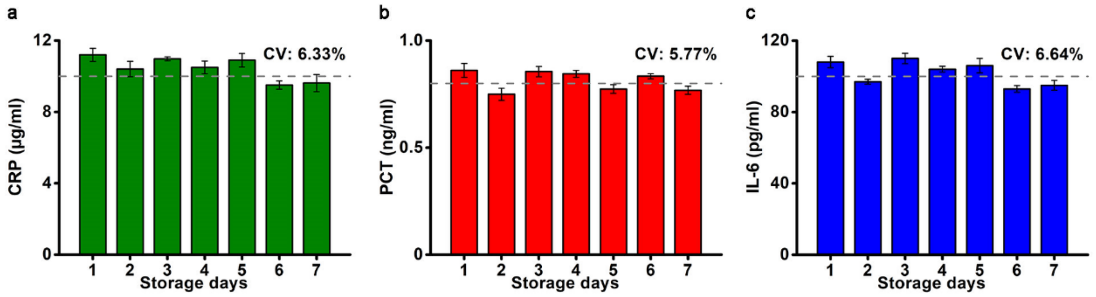

Fig. S8 Storage stability of CRP, PCT, and IL-6. **a** Results of detecting 10 μg/ml CRP in WMC-MDP, which have been stored for 1-7 days. **b** Results of detecting 0.8 ng/ml PCT in WMC-MDP, which have been stored for 1-7 days. **c** Results of detecting 100 pg/ml IL-6 in WMC-MDP, which have been stored for 1-7 days. CRP c-reactive protein, PCT procalcitonin, IL-6 interleukin-6, CV coefficients of variation, WMC-MDP wave-shaped microfluidic chip assisted multiplexed detection platform
